# Analgesic effects of different patient-controlled intravenous analgesia infusion modes post cesarean section under multimodal analgesia: a retrospective cohort study

**DOI:** 10.3389/fmed.2026.1854392

**Published:** 2026-06-18

**Authors:** Lin Chen, Yang Wang, Shanshan Ye, Tao Xu, Ding Huang

**Affiliations:** 1 Department of Central Operating Room, International Peace Maternity and Child Health Hospital, School of Medicine, Shanghai Jiao Tong University, Shanghai, China; 2 Shanghai Key Laboratory of Embryo Original Diseases, Shanghai, China; 3 Department of Anesthesiology, International Peace Maternity and Child Health Hospital, School of Medicine, Shanghai Jiao Tong University, Shanghai, China; 4 Department of Obstetrics, International Peace Maternity and Child Health Hospital, School of Medicine, Shanghai Jiao Tong University, Shanghai, China

**Keywords:** obstetrical analgesia, opioid, pain management, patient controlled analgesia, post cesarean section

## Abstract

**Background:**

Background infusion is frequently employed in postoperative patient controlled intravenous analgesia. This study aimed to compare the analgesic efficacy of patient-controlled intravenous analgesia with and without background infusion for parturients receiving multimodal analgesia incorporating liposomal bupivacaine transversus abdominis plane block post cesarean delivery.

**Methods:**

A total of 250 parturients who underwent elective cesarean section with multimodal postoperative analgesia between January 2025 and December 2025 were divided into background infusion and no background infusion group. Parturients received epidural hydromorphone, bilateral transversus abdominis plane block with 266 mg liposomal bupivacaine and routine oral acetaminophen. Patient controlled intravenous analgesia settings differed in background infusion (1.0 vs. 0 ml/h), with the same bolus (2.0 ml) and lockout interval (10 min); the solution contained oxycodone or hydromorphone. Patient controlled analgesia demands, intravenous morphine equivalent dose of opioids, rest/ambulation pain scores, adverse reactions and analgesic effect were compared.

**Results:**

No significant differences were found in patient-controlled analgesia demands during 0–24 h (3 [0, 6] vs. 2 [1, 6], *p* = 0.829) and 24–48 h (5 [1, 12] vs. 4 [1, 10], *p* = 0.303) or in rest/ambulation pain scores during 0–24 and 24–48 h between two groups. Background infusion group had higher 48-h intravenous morphine equivalent doses (3.5 [0.7, 8.5] mg vs. 54.6 [44.9, 62.0] mg, *p* < 0.001) postoperatively.

**Conclusion:**

In parturients receiving multimodal analgesia consisting of transversus abdominis plane block with liposomal bupivacaine, epidural hydromorphone and routine oral acetaminophen, the addition of background infusion to patient-controlled intravenous analgesia failed to enhance analgesic efficacy and increased opioid exposure. Patient-controlled intravenous analgesia without background infusion may be considered in such cases.

## Introduction

Cesarean section is associated with moderate-to-severe postoperative pain (1); poorly managed pain may develop into chronic pain (2), impair mother-child bonding (1), and negatively impact maternal psychological well-being (3). Therefore, adequate control of post-cesarean section pain is critically important.

Current guidelines (1, 4, 5) all recommend multimodal analgesia for postoperative pain management post cesarean section, including neuraxial opioids, routine oral acetaminophen or non-steroidal anti-inflammatory analgesics, abdominal nerve block, as well as intravenous or oral opioid analgesia.

With the emergence of the long-acting local anesthetic liposomal bupivacaine, multimodal analgesia combined with transversus abdominis plane (TAP) block has been proven to provide longer and more satisfactory analgesic effect after cesarean delivery (6–10). Therefore, for multimodal analgesia incorporating liposomal bupivacaine TAP block, it is necessary to re-explore whether a background infusion is required in patient controlled intravenous analgesia (PCIA) regimens. This study hypothesizes that in multimodal analgesia incorporating liposomal bupivacaine TAP block, intravenous analgesia with background infusion provides no superior analgesic efficacy yet significantly increases opioid consumption post cesarean section.

## Materials and methods

### Study design and data selection

This was a retrospective study, which mainly used the postoperative analgesia data of parturients who received multimodal analgesia regimens after elective cesarean delivery between January 2025 and December 2025 in International Peace Maternity and Child Health Hospital (a tertiary Class A obstetrics and gynecology hospital). This study was approved by the Ethics Committee of the International Peace Maternity and Child Health Hospital (Approval No.: GKLW-A-2024-032-01). Since this was a retrospective study, which mainly conducted comparative analysis on postoperative analgesia data, and all analgesia regimens in the study were performed in accordance with the routine of our hospital, informed consent was not obtained from the parturients in the study.

Inclusion criteria: parturients aged 20–40 years old, American Society of Anesthesiology physical status II, singleton pregnancy, full-term (gestational age 37–42 weeks), who underwent elective cesarean section, received TAP block with liposomal bupivacaine, epidural hydromorphone and routine oral acetaminophen postoperatively. All parturients agreed to the use of analgesia pump after informed consent. Other postoperative multimodal analgesia regimen included epidural hydromorphone or routine oral acetaminophen postoperatively. Exclusion criteria included parturients with severe postpartum hemorrhage (postoperative 24-h hemorrhage exceeding 1000 ml), contraindications to neuraxial anesthesia, allergy to study medications (liposomal bupivacaine, ropivacaine, hydromorphone, oxycodone, acetaminophen) or a history of drug abuse, cases with incomplete postoperative analgesia records, and those administered intravenous analgesics other than μ-receptor agonists during the postoperative period.

### Postoperative analgesic methods

1. Liposomal bupivacaine TAP block: According to routine practice, after the end of surgery, the anesthesiologist performed bilateral lateral TAP block under ultrasound guidance. The drug used was 266 mg liposomal bupivacaine (241217BL; Hengrui Medical Co., Ltd., Lianyungang, Jiangsu, China), which was diluted to 40 ml with normal saline, and 20 ml was injected on each side.
2. Epidural analgesia: According to routine practice, after the fetus was delivered and before the epidural catheter was removed at the end of surgery, 0.6 mg hydromorphone (AB40505221, Renfu Medical Co., Ltd., Yichang, Hubei, China) in a volume of 6 ml was administered through the epidural catheter.
3. Postoperative oral acetaminophen: One box of acetaminophen (2212007, Chelonia Healthcare Ltd., Hampshire, UK) was given to the parturient before the end of surgery, and she was instructed to take one tablet orally by herself immediately after returning to the ward, and then take one tablet orally every 6 h until 72 h postoperatively.
4. PCIA: An PCIA device was connected after the end of surgery. Intravenous analgesia could be divided into two analgesic modes: with background dose and without background dose. The drugs used were oxycodone (NQK24I02, Nhwa Pharmaceutical Co., Ltd., Xuzhou, Jiangsu, China) or hydromorphone according to the clinical routine for post cesarean analgesia of our department, with solution concentrations of 0.5 mg/ml and 0.1 mg/ml, respectively. When the background dose was used, the PCIA was set as follows: background dose 1 ml/h, patient controlled analgesia (PCA) dose 2 ml per time, lockout interval 10 min; when no background dose was used, the PCIA was set as: background dose 0 ml/h, PCA dose 2 ml per time, lockout interval 10 min. After connecting the analgesia pump, the parturient was informed of the usage method of the analgesia pump, and it was emphasized that if the numerical rating scale (NRS) score was >3 at rest, the parturient could press the PCA button of the analgesia pump for rescue analgesia.

### Collection of postoperative analgesic effect and maternal adverse reactions

At approximately 9:00 AM, 1:00 PM, and 5:00 PM, an anesthetic nurse followed up and retrieved the analgesic device. The main tasks were to record the maternal worst rest and ambulation pain in 24 and 48 h postoperatively, feedback and treatment of adverse reactions, operation status of the analgesia pump (including whether the analgesia pump was running, the number of PCA presses, and the total amount of drugs used), guide and advise the parturient on the use of the analgesia pump and oral analgesic drugs, and collect the parturient’s feedback.

### Extracted indicators and primary outcome measure

The indicators extracted in this study included maternal general conditions, maternal analgesic status, and adverse reactions. General conditions included maternal age, height, weight, gravidity, parity, gestational age, intraoperative use of uterotonic agents, operation time, and intraoperative blood loss, drugs for PCIA which were mainly obtained from the DoCare Clinical Anesthesia Information System (Medical System Medical Technology Co., Ltd., Suzhou, Jiangsu, China). Maternal analgesic status included total amount of intravenous opioids used, rest pain, ambulation pain, number and percentage of PCA presses during 0–24 and 24–48 h postoperatively, and satisfaction with analgesic effect; analgesic adverse reactions including dizziness, nausea and vomiting, pruritus, sedation level, and urinary retention were all extracted from the follow-up records of anesthetic nurses. Sedation level was assessed by anesthetic nurses using a 3-point sedation scale, with grades defined as awake, drowsy, and asleep (11). Postoperative urinary retention was diagnosed by the presence of voiding symptoms (incomplete emptying, straining, voiding difficulty) or the requirement for Foley catheter reinsertion after catheter removal (12).

For the comparability of the two intravenous opioids used for analgesia, the total doses of the two opioids were converted to intravenous morphine equivalents (IME) doses. Intravenous oxycodone was converted to intravenous morphine at a ratio of 0.71:1, and intravenous hydromorphone was converted to intravenous morphine at a ratio of 1:7 (13).

The primary outcome measure of this study was the number of PCA presses during 0–24 h and 24–48 h in no background infusion (NBI) group and background infusion (BI) group postoperatively.

### Sample size calculation and statistical analysis

Based on the Mann-Whitney U test for two independent groups. According to a previous study (8), when the same analgesic regimen as that in NBI group was adopted, the median and interquartile range of PCA presses at 24 h after cesarean section were 2 (0, 5). Assuming that the median PCA presses in BI group of this study decreased by 1, with an effect size (Cohen’s d) of 0.5, a two-sided type I error rate of 0.05, and a power of 0.80, a sample size of 85 patients per group was required. The sample size adopted in this study was larger than the sample size calculated. Sample size calculation was performed using PASS 2021 software (NCSS LLC., Kaysville, UT, USA).

Continuous data were presented as the mean ± SD or median and interquartile range (IQR), and groups were compared using *t*-tests or Mann–Whitney U tests, as appropriate. Categorical variables were presented as frequencies and percentages and groups were compared using chi-square or Fisher’s exact tests. The effect sizes for outcomes related to post-operative analgesia effect were calculated using the “effsize” package Cliff’s delta was adopted to calculate the effect size of continuous and ordinal variables. A value close to 0 indicates negligible effect, while values approaching −1 or +1 represent a large effect size. Median difference was used to assess effect size for continuous variables, and risk difference was applied for categorical variables. Violin Plots of worst NRS for rest and ambulation pain in 0–24, and 24–48 h postoperatively were generated for each group using the “ggplot2” R software package. These statistical analyses were performed using R 4.4.1 (R Foundation for Statistical Computing, Vienna, Austria). Statistical significance was defined as a two-tailed *p*-value < 0.05.

## Results

**FIGURE 1:**
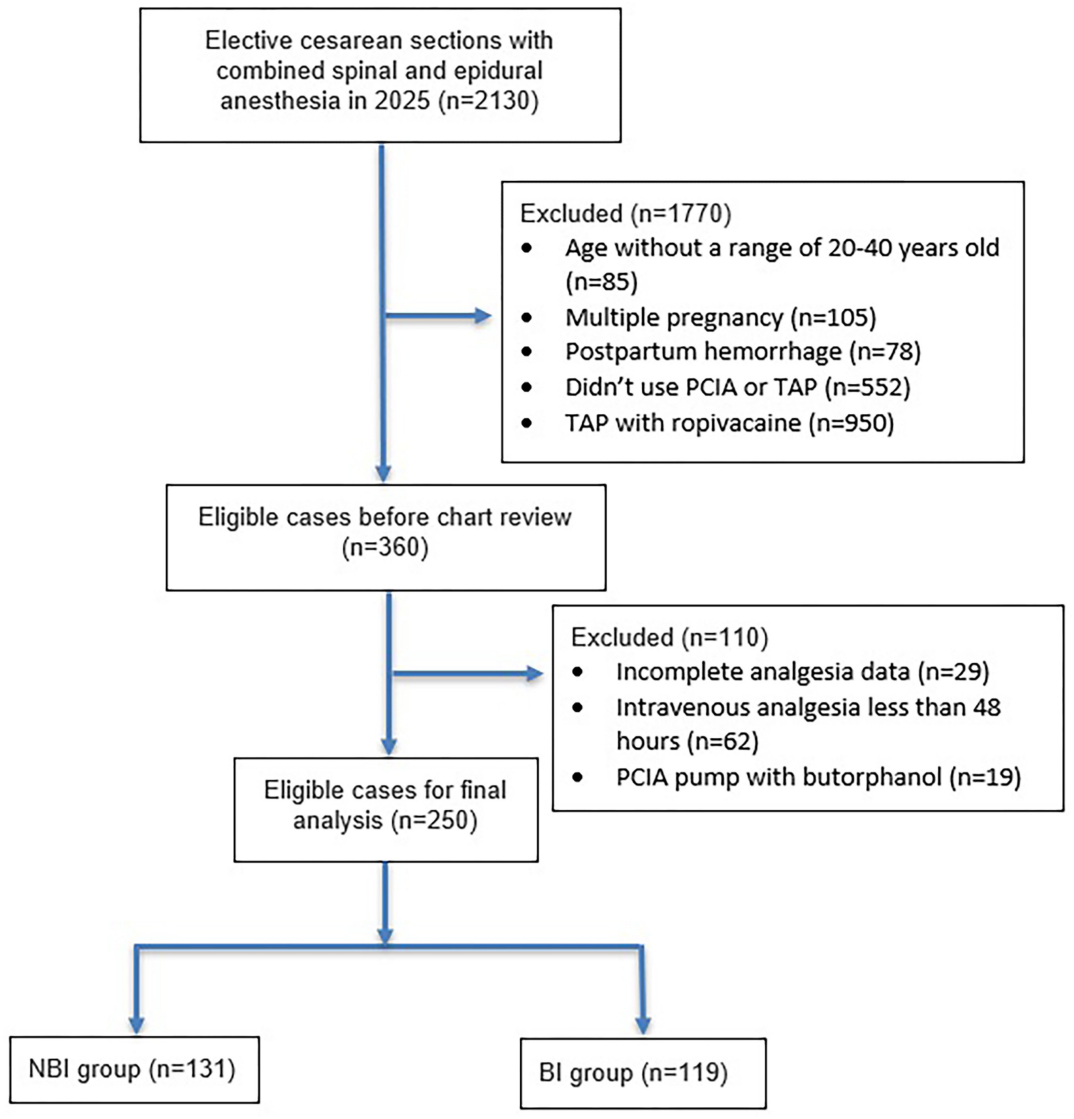
Flow diagram of the study.

A total of 2130 parturients undergoing elective cesarean section under combined spinal-epidural anesthesia (CSEA) with ASA II status in 2025 were extracted from the DoCare Anesthesia Record System using the keywords: cesarean section, combined spinal-epidural anesthesia, and ASA II. After excluding 85 parturients aged <20 or >40 years, 105 parturients with multiple gestation, 78 parturients with postpartum hemorrhage following cesarean section, 552 parturients without TAP block or PCIA analgesia, and 950 parturients receiving ropivacaine for TAP block, 360 parturients met the initial inclusion criteria. Postoperative analgesia data were reviewed in these 360 parturients. Among them, 29 had incomplete data and 62 had insufficient intravenous analgesia duration (<48 h). In addition, butorphanol acts as a μ receptor agonist-antagonist and kappa-receptor agonist. To reduce the bias caused by conversion to IME dose, 19 patients receiving intravenous butorphanol analgesia were excluded from the analysis. Finally, 250 parturients were included into the analysis, including 131 in the NBI group and 119 in the BI group (Figure 1).

Table 1 shows the baseline demographics and clinical characteristics of the parturients between two groups. There were no significant differences in maternal age, height, weight, gravidity, parity, gestational age, baby birth weight, intraoperative dosage of uterotonic agents, operation time, blood loss, and the percentage of parturients who received epidural hydromorphone or timely oral acetaminophen for postoperative analgesia. All parturients in the NBI group received oxycodone for PCIA, while in the BI group, the usage percentages of oxycodone, hydromorphone were 49.6% and 50.4%, respectively, with a significant difference in the composition between the two groups (*p* < 0.001).

**TABLE 1:** Characteristics and baseline assessments.

Maternal characteristics	NBI group (*n* = 131)	BI group (*n* = 119)	*P*-value
Age, year	33.0 ± 4.1	33.4 ± 4.3	0.454
Height, cm	163.5 ± 5.4	162.4 ± 4.0	0.070
Weight, kg	71.5 ± 8.8	72.8 ± 9.4	0.254
Gravidity	1 (1, 2)	1 (1, 2)	0.150
Parity	0 (0, 0)	0 (0, 0)	0.165
Gestational age, week	38.8 ± 1.1	38.1 ± 3.9	0.050
Birth weight, g	3286 ± 366	3208 ± 460	0.144
Intraoperative uterotonic agent used			
1	108 (82.4%)	88 (73.9%)	
2	20 (15.3%)	27 (22.7%)	
3	3 (2.3%)	4 (3.4%)	0.265
Surgical time, min	44.7 ± 37.6	51.0 ± 15.9	0.197
Blood loss, ml	214 ± 56	229 ± 116	0.195
Post-operative analgesia			
TAP with liposomal bupivacaine, *n* %	131 (100%)	119 (100%)	1.000
Epidural hydromorphone, *n* %	131 (100%)	119 (100%)	1.000
Timely oral acetaminophen, *n* %	131 (100%)	119 (100%)	1.000
Intravenous opioid agent used, n %			
Oxycodone	131 (100%)	59 (49.6%)	<0.001
Hydromorphone	0 (0)	60 (50.4%)	

Data are presented as mean ± standard deviation, median (interquartile range) or number (percentage) as appropriate.

**FIGURE 2:**
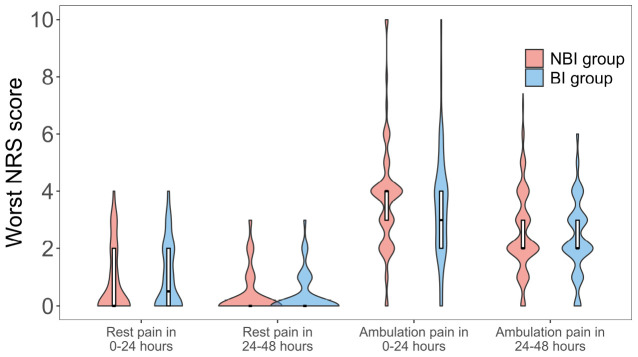
Violin plots of worst rest and ambulation NRS pain scores in post-operative 0–24, 24–48 h. The worst rest and ambulation NRS pain scores were comparable between NBI and BI group in post-operative 0–24, 24–48 h. NRS, numeric rating scale; NBI, no background infusion; BI, background infusion.

Postoperative analgesic status included all 250 cases is shown in Table 2. The number of PCA presses during 0–24 h and 24–48 h was (3 [0, 6] vs. 2 [1, 6], *p* = 0.829) and (5 [1, 12] vs. 4 [1, 10], *p* = 0.303), respectively, with no median differences between the two groups. Total oxycodone consumption dose was 2.5 (0.5, 6.0) mg in NBI group and 33.0 (26.0, 44.0) mg in BI group, total hydromorphone consumption dose was 8.0 (7.0, 9.0) mg in BI group. The IME dose of opioids used for PCIA in the NBI group was significantly lower than that in the BI group (3.5 [0.7, 8.5] mg vs. 54.6 [44.9, 62.0] mg, *p* < 0.001). The percentages of parturients using PCA on the first and second postoperative days were 71.0% and 79.4% in the NBI group, while those in the BI group were 79.0% and 80.7%, respectively, with no differences between groups. There was also no difference in analgesia satisfaction between the two groups. The worst postoperative rest and ambulation pain scores were comparable in 0–24 and 24–48 h (Figure 2).

**TABLE 2:** Outcomes related to post-operative analgesia effect.

Outcomes	NBI group (*n* = 131)	BI group (*n* = 119)	Difference	*P*-value	Effect size with 95% CI
Primary outcomes					
PCA times in 0–24 h	3 (0, 6)	2 (1, 6)	−1 (−2.5, 0.5)	0.829	−0.016 (−0.159 to 0.129)
PCA times in 24–48 h	5 (1, 12)	4 (1, 10)	−1 (−3.4, 1.4)	0.303	0.075 (−0.062 to 0.214)
Secondary outcomes					
Oxycodone, mg	2.5 (0.5, 6.0)	33.0 (26.0, 44.0)	/	/	/
Hydromorphone, mg	/	8.0 (7.0, 9.0)	/	/	/
IME dose for 48 h, mg	3.5 (0.7, 8.5)	54.6 (44.9, 62.0)	51.1 (47.1, 55.0)	<0.001	−1.000 (−1.000 to −1.000)
PCA in 0–24 h, *n* (%)	93 (71.0%)	94 (79.0%)	0.08 (−0.19, 0.03)	0.191	0.185 (−0.063 to 0.433)
PCA in 24–48 h, *n* (%)	104 (79.4%)	96 (80.7%)	0.01 (−0.12, 0.09)	0.924	0.032 (−0.216 to 0.280)
5-Likert scores of analgesia effect	5 (5, 5)	5 (5, 5)	0 (0, 0)	0.314	−0.053 (−0.157 to 0.044)

Data are presented as median (interquartile range) or number (percentage) as appropriate. Median difference was used to assess effect size for continuous variables, and risk difference was applied for categorical variables. PCA, patient-controlled analgesia; IME, intravenous morphine equivalent; 95% CI, 95% confidence interval.

Table 2 presents the sensitivity analysis comparing 59 oxycodone-treated cases in the BI group with the 131 oxycodone cases in the NBI group. The number of PCA presses during 0–24 h and 24–48 h was (3 [0, 6] vs. 3 [1, 8], *p* = 0.369) and (5 [1, 12] vs. 5 [1, 13], *p* = 0.911), respectively, with no median differences between the two groups. The total oxycodone used in NBI group was significantly lower than that in the BI group (2.5 [0.5, 6.0] mg vs. 33.0 [26.0, 44.0] mg, *p* < 0.001). The IME dose of oxycodone used for PCIA in the NBI group was significantly lower than that in the BI group (3.5 [0.7, 8.5] mg vs. 46.5 [36.7, 62.0] mg, *p* < 0.001). The percentages of parturients using PCA on the first and second postoperative days were 71.0% and 79.4% in the NBI group, while those in the BI group were 78.0% and 76.3%, respectively, with no differences between groups. There was also no difference in analgesia satisfaction between the two groups. The results of sensitivity analysis were highly consistent with those of the overall sample.

Analgesia-related adverse reactions of the two groups are shown in Table 3. The incidence of dizziness, nausea and vomiting, pruritus, drowsiness and urinary retention were comparable between the two groups postoperatively.

**TABLE 3:** Side effects of post-operative analgesia.

Side effects	NBI group (*n* = 131)	BI group (*n* = 119)	*P*-value
Dizziness, *n* (%)	10 (7.6%)	12 (10.1%)	0.495
Nausea and vomiting, *n* (%)	13 (9.9%)	10 (8.4%)	0.678
Pruritus, *n* (%)			0.917
None	96 (73.3%)	89 (74.8%)	
Mild	31 (23.7%)	28 (23.5%)	
Moderate	2 (1.5%)	1 (0.8%)	
Severe	2 (1.5%)	1 (0.8%)	
Drowsiness, *n* (%)	0 (0%)	0 (0%)	1.000
Urinary retention, *n* (%)	12 (9.2%)	12 (10.1%)	0.804

Data are presented as number (percentage).

## Discussion

The results demonstrated that under the multimodal analgesia regimen consisting of liposomal bupivacaine TAP block, epidural hydromorphone and routine oral acetaminophen post cesarean section, no significant difference was found in PCA pressing times between the NBI and BI groups at 0–24 h and 24–48 h postoperatively, and the analgesic efficacy was similar between the two groups, while the total amount of intravenous opioids used in BI group was significantly higher than that in the NBI group.

Transversus abdominis plane block with liposomal bupivacaine has been demonstrated to provide prolonged and satisfactory analgesia for postoperative pain management after cesarean section (6–10). Postoperative multimodal analgesia dominated by liposomal bupivacaine TAP block and neuraxial opioid administration post cesarean section has been validated in several studies (6, 8). However, in such multimodal analgesia regimens, it remains uninvestigated to date whether intravenous analgesia is necessary, and if so, what mode of intravenous analgesia should be adopted.

Yefet et al. had shown that the proportion of patients with an NRS score ≥ 4 within 24 h after surgery was close to or exceeds 50%, whether receiving oral analgesia or intrathecal morphine analgesia (14). A study by Hatter et al. indicated that even with 100 μg of intrathecal morphine used postoperatively, the proportion of parturients requiring oral oxycodone after cesarean section was as high as 73.2% (15). In this study, the percentages of parturients using rescue PCA on the first and second postoperative days were 71.0% and 79.4% in the NBI group, while those in the BI group were 79.0% and 80.7%, respectively. Therefore, it is very necessary to set up rescue analgesia after cesarean section. The rescue method in this study was an intravenous analgesia regimen, which has a faster onset of action, no gastrointestinal first-pass effect, and is more suitable for breakthrough pain and moderate to severe pain. Moreover, with the use of the PCA mode in the current study showed the parturients’ pain relief is faster and the incidence of side effects is not high.

It was previously believed that the use of a background dose for analgesia could maintain a stable blood drug concentration and analgesic effect (16). However, for long-acting opioids, the additive effect of this action is prone to drug overdose, leading to an increased incidence of adverse reactions (16, 17). In 2016, the American Pain Society recommended that for patients who intolerant to opioids, routine use of a background dose is not recommended when using PCIA postoperatively (18). Our current study further confirmed that under the premise of multimodal analgesia incorporating liposomal bupivacaine TAP block, the use of a background dose cannot reduce the number of PCA presses required by parturients, nor can it produce a better analgesic effect, and the usage of opioids is significantly increased.

Postoperative pain after cesarean section is different from pain after other surgeries, consisting of surgical incision pain and uterine contraction pain (19). As a dual μ and κ opioid receptor agonist, oxycodone can effectively improve postoperative incision pain through its μ receptor agonist effect, and significantly reduce the occurrence of postoperative visceral pain through its κ receptor agonist effect (20). And it was reported to provide more satisfactory analgesia effect compared with sufentanil and fentanyl (21). Oral oxycodone also produces effective analgesia, with superior efficacy when administered as a combination preparation with acetaminophen (14, 20, 22, 23). More cost-benefit analyses are needed to determine the advantages and disadvantages of the two different administration routes in terms of management cost and drug price to produce analgesic benefits.

The strength of this study is the first to investigate whether a background infusion is required for PCIA following multimodal analgesia regimen consisting of liposomal bupivacaine TAP block, epidural hydromorphone and routine oral acetaminophen post cesarean section. With a sufficient sample size, it provides evidence for clinical practice.

The limitations of this study include being a single-center retrospective study, which may require more randomized controlled studies for further confirmation. In addition, the departmental medication policy and drug price changed from 2024 to 2025, together with individual prescribing preference among anesthesiologists, leading to uneven distribution of opioid agents between groups. Thus, practice transition over time, together with potential physician-specific variations and time-related confounding factors, should be acknowledged as study limitations. Third, the discrepancies described in the second point resulted in heterogeneity in medication use between the two groups. However, baseline characteristics were comparable between the two groups and we converted the total consumption of the two μ receptor agonists opioids used in two groups to IME to minimize this confounding effect. Furthermore, an independent sensitivity analysis was performed on cases receiving intravenous oxycodone analgesia in both groups. Admittedly, the oxycodone-only sensitivity analysis only supports the robustness of the findings and cannot completely eliminate confounding bias. Finally, residual urine volume was not measured by ultrasonography, and maternal subjective feelings were adopted as the diagnostic criterion for urinary retention in the study, which inevitably brings certain limitations.

In conclusion, for parturients receiving TAP block with liposomal bupivacaine combined with multimodal analgesia consisting of epidural hydromorphone and routine oral acetaminophen post cesarean section, adding background infusion to PCIA fails to produce extra analgesic benefits and increases opioid consumption. Accordingly, PCIA without background infusion may be considered in such cases.

## Data Availability

The raw data supporting the conclusions of this article will be made available by the corresponding author (dingding123hos@163.com), without undue reservation.
